# Structural Variation at a Disease Mutation Hotspot: Strategies to Investigate Gene Regulation and the 3D Genome

**DOI:** 10.3389/fgene.2022.842860

**Published:** 2022-03-25

**Authors:** Alexandra Boyling, Gonzalo Perez-Siles, Marina L. Kennerson

**Affiliations:** ^1^ Northcott Neuroscience Laboratory, ANZAC Research Institute, Sydney, NSW, Australia; ^2^ Sydney Medical School, University of Sydney, Sydney, NSW, Australia; ^3^ Molecular Medicine Laboratory, Concord Repatriation General Hospital, Sydney, NSW, Australia

**Keywords:** structural variation (SV), long-range gene regulation, 3D genome, interchromosomal insertions, Xq27.1 palindrome

## Abstract

A rare form of X-linked Charcot-Marie-Tooth neuropathy, CMTX3, is caused by an interchromosomal insertion occurring at chromosome Xq27.1. Interestingly, eight other disease phenotypes have been associated with insertions (or insertion-deletions) occurring at the same genetic locus. To date, the pathogenic mechanism underlying most of these diseases remains unsolved, although local gene dysregulation has clearly been implicated in at least two phenotypes. The challenges of accessing disease-relevant tissue and modelling these complex genomic rearrangements has led to this research impasse. We argue that recent technological advancements can overcome many of these challenges, particularly induced pluripotent stem cells (iPSC) and their capacity to provide access to patient-derived disease-relevant tissue. However, to date these valuable tools have not been utilized to investigate the disease-associated insertions at chromosome Xq27.1. Therefore, using CMTX3 as a reference disease, we propose an experimental approach that can be used to explore these complex mutations, as well as similar structural variants located elsewhere in the genome. The mutational hotspot at Xq27.1 is a valuable disease paradigm with the potential to improve our understanding of the pathogenic consequences of complex structural variation, and more broadly, refine our knowledge of the multifaceted process of long-range gene regulation. Intergenic structural variation is a critically understudied class of mutation, although it is likely to contribute significantly to unsolved genetic disease.

## Introduction

Charcot-Marie-Tooth (CMT) neuropathy refers to a group of inherited conditions characterized by the length-dependent degeneration of both motor and sensory neurons within the peripheral nervous system. In the absence of a cure for this debilitating condition, individuals with CMT present with varying degrees of distal muscle wasting and sensory impairment, leading to chronic disability (Timmerman et al., 2014). This disease is highly genetically heterogeneous, with more than 1,000 causative mutations reported in over 80 genes (Timmerman et al., 2014). These mutations range from single nucleotide changes to complex copy number variations, however, to date there has only been one interchromosomal insertion associated with CMT. A rare X-linked form of CMT, CMTX3, is caused by the insertion of 78 kb of DNA originating from chromosome 8q24.3 into an intergenic region of the CMTX3 locus on chromosome Xq27.1 (Brewer et al., 2016).

Interestingly, the CMTX3 insertion occurs within a quasi-palindromic sequence on chromosome Xq27.1 which is a recurring hotspot for genomic rearrangements. To date, eight additional disease phenotypes have been associated with complex insertions, or insertion-deletions, occurring at this specific locus (Bowl et al., 2005; Zhu et al., 2011; Chou, 2012; DeStefano et al., 2013; Bunyan et al., 2014; Haines et al., 2015; Taylor et al., 2015; Middelkamp et al., 2019; Si et al., 2019; Boschann et al., 2022). For simplicity, we will collectively refer to these mutations as ‘the disease-associated insertions at Xq27.1’ with Table 1 providing additional information regarding the complexity of these genomic rearrangements. This genomic hotspot resides within a gene desert, with approximately 82 kb of non-coding DNA separating the palindrome from the nearest coding gene (Zhu et al., 2011). Most researchers reporting these novel rearrangements hypothesize that they cause disease by disrupting the regulation of nearby genes (Zhu et al., 2011; Haines et al., 2015; Brewer et al., 2016; Middelkamp et al., 2019; Si et al., 2019; Boschann et al., 2022). However, it has proven very challenging to identify the specific gene(s) being dysregulated by the various insertions, and therefore, the pathogenic mechanisms underlying many of these diseases remain unsolved.

**TABLE 1:** Disease phenotypes associated with insertions (+/− deletions) at the Xq27.1 mutational hotspot. Genomic coordinates are based on the GRCh37/hg19 reference sequence.

Disease phenotype	Additional phenotypic features	Insertion sequence	Insertion size and orientation	Genes contained in insertion		Additional accompanying rearrangements at Xq27.1		References
X-linked hypoparathyroidism (OMIM: #307700)	N/A	2p25.3	∼340 kb (Direct)	*SNTG2* (partial)		∼25 kb deletion		Bowl et al. (2005)
X-linked hypoparathyroidism (OMIM: #307700)	N/A	2p25.3 (Chr2: 856,644-903,578)	∼47 kb (Direct)	Non-coding DNA only		∼1.5 kb deletion		Taylor et al. (2015)
						3 bp insertion		
X-linked congenital generalized hypertrichosis (OMIM: #307150)	N/A	4q31.22-q31.23 (Chr4: 148,464,548-148,764,583)	∼300 kb (Inverted)	*PRMT9* (full)		25 bp insertion		Zhu et al. (2011)
				*TMEM184C* (full)				
				*ARHGAP10* (partial)				
				*EDNRA* (partial)				
						7 bp deletion^a^		
X-linked congenital generalized hypertrichosis (OMIM: #307150)	Scoliosis, spina bifida	5q35.3 (Chr5: 177,831,590-177,957,166)	∼126 kb (Direct)	*COL23A1* (partial)		∼1.3 kb deletion		Zhu et al. (2011)
						124 bp insertion (from Xq28)		
						2 bp insertion		
X-linked congenital generalized hypertrichosis (OMIM: #307150)	Deafness, dental/palate anomalies	6p21.2 (Chr6: 39,786,474-39,397,468)	∼389 kb (Inverted)	*KIF6* (partial)		56 bp insertion (from 3q21.2)		DeStefano et al. (2013)
						14 bp insertion		
						2 bp deletion		
				*DAAM2* (partial)				
X-linked congenital bilateral ptosis (OMIM: %300,245)	N/A	1p21.3 (Chr1: 97,886,267-98,006,168)	∼120 kb (Direct)	*DPYD* (partial)		7 bp duplication		Bunyan et al. (2014)
						427 bp duplication		
Bilateral anophthalmia and XX sex reversal	Neurological abnormalities, orbital teratoma	9q21	640 kb (Direct)	*TRPM3* (partial)		4 bp inversion		Chou, (2012)
						4 bp deletion		
XX male sex reversal	N/A	1q25.2—1q25.3 (Chr1: 180,243,986-181,017,783)	∼774 kb (Direct)	ACBD6 (full)		9 bp insertion		Haines et al. (2015)
				XPR1 (full)		4 bp deletion		
				KIAA1614 (full)				
				STX6 (full)				
				OVAAL (full)				
				MIR3121 (full)				
				LHX4 (partial)				
				MR1 (partial)				
Charcot-Marie-Tooth neuropathy, X-linked recessive, 3 (OMIM: #302802)	N/A	8q24.3 (Chr8:145,768,312–145,848,158)	∼78 kb (Direct)	*ARHGAP39* (partial)		19 bp insertion (from 12q13.12)		Brewer et al. (2016)
						12 bp inversion		
						1 bp deletion		
						1 bp single nucleotide variant		
Rare X-linked recessive compound phenotype (genu varum, cubitus valgus, everted lips)	N/A	Xp22.33/Yp11.32 (ChrX: 628,417-733,365/ChrY: 578,417-683,365)	∼105 kb (Direct)	Non-coding DNA only		N/A		Si et al. (2019)
Multiple congenital abnormalities	N/A	9p22.3-9p22.2 (Chr9: 16,489,097-16,659,203)	∼170 kb (Inverted)	*BNC2* (partial)		4 bp deletion^a^		Middelkamp et al. (2019)
X-linked congenital bilateral laryngeal abductor paralysis (Plott syndrome, OMIM: 308850)	N/A	10q21.3 (Chr10: 65,948,754-66,352,777)	∼404 kb (Inverted)	Non-coding DNA only		∼10 kb deletion		Boschann et al. (2022)
						59 bp insertion (from 10q21.3)		
						7 bp insertion		
						2 bp insertion		

a Deletion has been inferred based on the reported coordinates of the insertion breakpoints.

This challenge has largely arisen from our incomplete understanding of the non-coding genome, which has historically been overlooked and considered “junk DNA”. Efforts of The ENCODE Project have provided fundamental resources to highlight the role of non-coding DNA in transcriptional regulation (ENCODE Project Consortium, 2012), however, there is still much to learn about the many complex processes controlling gene expression profiles at a cell-specific level. Technical limitations have also prohibited researchers from effectively investigating the disease-associated insertions at Xq27.1, including limited access to relevant patient tissue and the difficulties of cloning large genomic rearrangements. Tissue specific gene dysregulation has been the hypothesized pathomechanism driving these diseases, and therefore, it is essential to use the appropriate tissue types when investigating each condition. For CMTX3, the challenge is gaining access to spinal cord and peripheral nerve tissues which are affected in patients (Brewer et al., 2016). Although the disease-associated insertions at Xq27.1 have been well-characterized at the sequence level, traditional cloning techniques cannot be used to generate cellular or animal models of disease. Strategies are therefore needed to generate relevant tissues whilst retaining patient-specific genomic rearrangements.

Since the discovery of the first disease phenotype caused by a complex insertion at Xq27.1 (Bowl et al., 2005), various advancements in technology have provided effective tools for overcoming many of these challenges. Perhaps most notably, improvements in cellular reprogramming technologies have made it possible to access patient-derived, disease-relevant tissue. Furthermore, improvements in transcriptome profiling and epigenome analyses can now facilitate a comprehensive interrogation of transcriptional organization and regulation in the appropriate tissue. To date, these valuable tools have not been utilized to investigate the different phenotypes associated with the insertions at Xq27.1.

With this in mind, the purpose of this review is to summarize the various phenotypes associated with complex rearrangements at Xq27.1 and highlight where recent technological advancements can be applied to gain insight into their underlying pathomechanisms. Using CMTX3 as the reference disease model, we propose an experimental approach that incorporates permutations of next-generation sequencing and induced pluripotent stem cell (iPSC) technologies to investigate how intergenic genomic rearrangements can produce disease. Understanding the contribution of intergenic structural variation to genetic disease is a growing area of interest, and the mutational hotspot at Xq27.1 provides a unique paradigm for increasing our knowledge of the complex, multi-faceted process of gene regulation.

## Insertions at the Xq27.1 Palindrome Cause Multiple Disease Phenotypes

Along with CMTX3, various other diseases are associated with complex insertions occurring at or near the palindrome sequence located at Xq27.1 (Table 1). The first of these, reported in 2005, was X-linked hypoparathyroidism (XL-HPT), arising from agenesis of the parathyroid glands (Bowl et al., 2005). This was caused by a complex genomic rearrangement involving a large insertion from chromosome 2p25.3 accompanied by a deletion at Xq27.1 (Bowl et al., 2005). Ten years later, another unrelated XL-HPT kindred was identified that also harbored an insertion from 2p25.3 and a deletion at Xq27.1 (Taylor et al., 2015). Intriguingly, despite both insertions originating from chromosome 2p25.3, the inserted sequences were not overlapping between the two kindreds (Gaynor et al., 2020). However, the Xq27.1 deletions that occurred in both families did partially overlap although differed considerably in size (Gaynor et al., 2020). Insertions at Xq27.1 also cause X-linked congenital generalized hypertrichosis (XL-CGH); a rare condition characterized by excessive hair growth (Zhu et al., 2011; DeStefano et al., 2013). Three unrelated XL-CGH kindreds have been reported, all harboring interchromosomal insertions of varying sizes that originate from different autosomes (Zhu et al., 2011; DeStefano et al., 2013). A phenotype of isolated bilateral ptosis (drooping eyelids) is associated with an insertion from chromosome 1p21.3 (Bunyan et al., 2014), whilst a different chromosome one insertion from 1q25.2-q25.3 causes XX male sex reversal (Haines et al., 2015). Interestingly, an XX male sex reversal phenotype was also observed in a patient with a *de novo* insertion from chromosome 9q21 as part of a complex disease phenotype that also included bilateral anophthalmia, orbital teratoma, and neurodevelopmental anomalies (Chou, 2012). An insertion involving the pseudoautosomal region of the sex chromosomes was found to cause a rare, undiagnosed phenotype consisting of multiple limb abnormalities (genu varum, cubitus valgus) and everted lips (Si et al., 2019), whilst a chromosome 9p22.3-p22.2 insertion was identified as a ‘variant of unknown significance’ (VUS) in a patient with multiple congenital abnormalities (Middelkamp et al., 2019). More recently, a large insertion originating from 10q21.3 was identified as the likely cause of Plott syndrome; a rare congenital disease characterized by bilateral laryngeal abductor paralysis (vocal cord paralysis) (Boschann et al., 2022).

**FIGURE 1:**
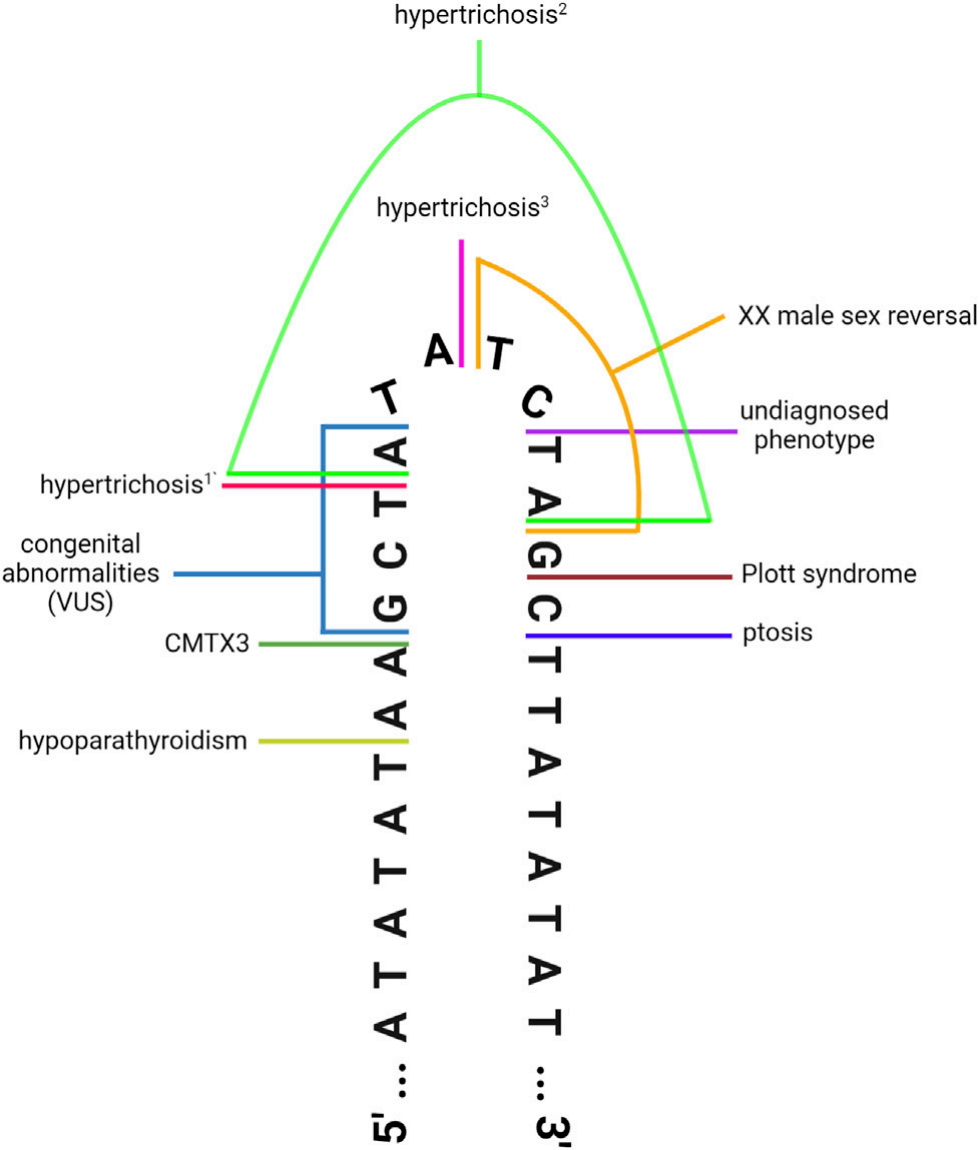
DNA-sequencing has revealed that the disease-associated insertions contain at least one breakpoint located toward the center of the 180 bp quasi-palindrome at Xq27.1. Illustration depicts the hairpin loop that is predicted to form at the center of the palindrome. Colored lines denote the position of the breakpoints for the insertions associated with hypoparathyroidism (Taylor et al., 2015), CMTX3 (Brewer et al., 2016), hypertrichosis^1,2^ (Zhu et al., 2011), hypertrichosis^3^ (DeStefano et al., 2013), XX male sex reversal (Haines et al., 2015), ptosis (Bunyan et al., 2014), Plott syndrome (Boschann et al., 2022), and a rare undiagnosed phenotype characterised by limb abnormalities and everted lips (Si et al., 2019). This schematic also depicts the breakpoints for a *de novo* insertion classified as a “variant of unknown significance” in a patient with congenital abnormalities (Middelkamp et al., 2019). Note that some of the insertions display additional breakpoints located outside this region (Zhu et al., 2011; Taylor et al., 2015; Boschann et al., 2022); see Table 1 for details. Figure has been adapted from Brewer et al. (2016) (Figure 4), originally published in *PLOS Genetics*, to incorporate additional disease-associated insertions that have been identified in recent years. Image created with BioRender.com.

**FIGURE 2:**
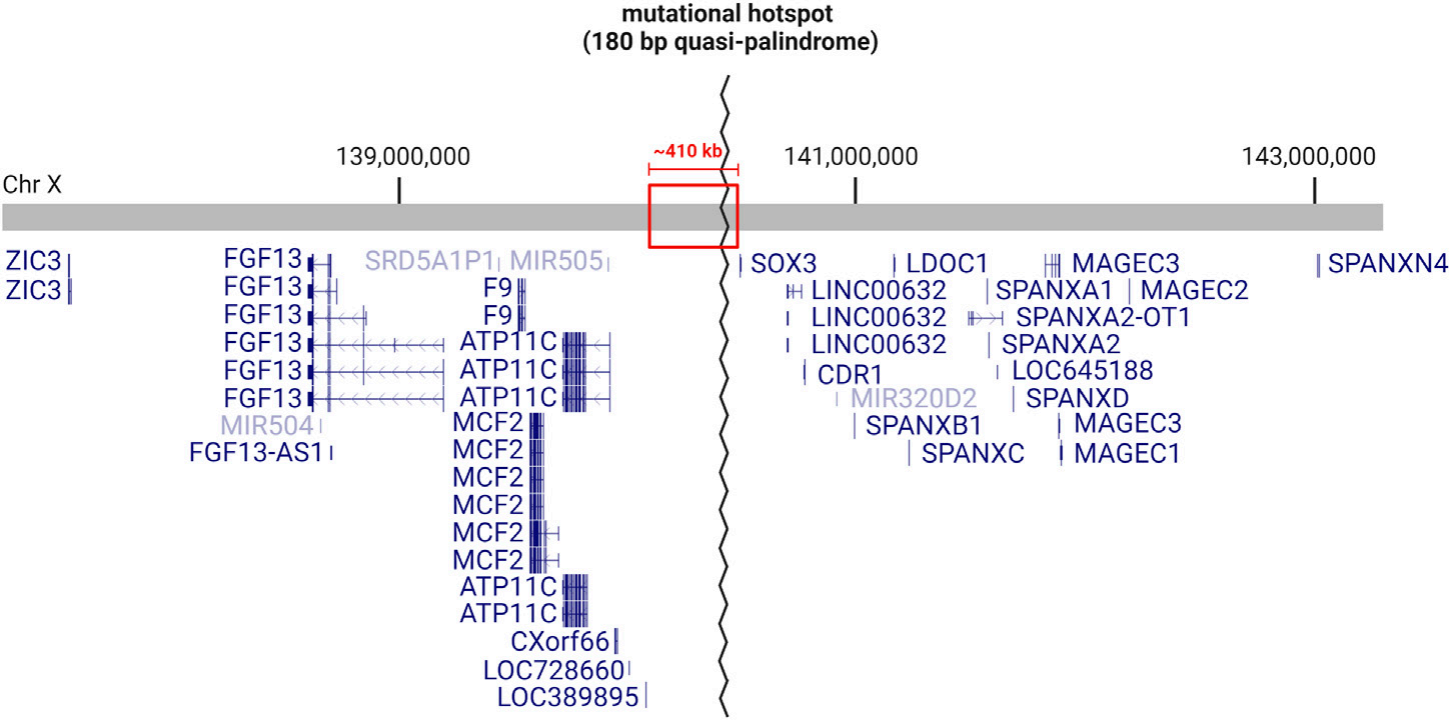
Genetic landscape of the mutational hotspot at chromosome Xq27.1. Diagram depicts the RefSeq curated genes located within ∼3 Mb on either side of the quasi-palindrome sequence, which acts as a hotspot for genomic rearrangements. The quasi-palindrome is situated within a ∼410 kb gene desert (red box), with 82 kb of non-coding sequence separating it from the closest coding gene (*SOX3*). Image created with BioRender.com using information obtained from UCSC Genome Browser (GRCh38/hg38).

Clearly, this region of chromosome Xq27.1 is prone to genomic rearrangements, likely due to the presence of the 180 bp quasi-palindrome sequence (ChrX: 139,502,865–139,503,044; hg19) which is predicted to form an unstable hairpin loop structure (Brewer et al., 2016). Accordingly, for the disease-associated insertions that have been mapped with genetic sequencing, at least one breakpoint was found to localize toward the center of this palindrome (Figure 1) (Brewer et al., 2016). Interestingly, this mutational hotspot localizes within a ∼410 kb gene desert with the nearest coding genes, *SOX3* and *LOC389895*, located approximately 82 and 329 kb away respectively (Figure 2) (Zhu et al., 2011; Brewer et al., 2016). Without any clear effect on coding DNA, defining the molecular mechanisms by which these intergenic structural variants produce disease remains a challenge.

## STRUCTURAL VARIATION CAN DISRUPT LONG-RANGE GENE REGULATION AND CAUSE GENETIC DISEASE

The disease-associated insertions at Xq27.1 are classified as structural variation (SV) mutations. SV is a broad term used to describe mutations that affect a minimum of 50 bp of DNA at a time (Sudmant et al., 2015). SV can vary in size ranging from 50 to millions of base pairs and are therefore capable of altering chromatin organization (Zhang et al., 2018). SV can include deletions and duplications (also known as copy number variation), as well as inversions, insertions, and chromosomal translocations (Yang et al., 2013). Complex combinations of the various SV subtypes can also occur (Sudmant et al., 2015). SVs involving duplications/deletions can exert pathogenicity by altering the dosage of genes contained within the rearranged DNA. CMT1A, the most common inherited neuropathy, is the quintessential example of a pathogenic genomic duplication, involving trisomy of the peripheral myelin protein 22 (*PMP22*) gene (Lupski et al., 1991; Matsunami et al., 1992; Patel et al., 1992; Timmerman et al., 1992; Valentijn et al., 1992). However, in the case of balanced SV, and SV affecting the non-coding genome, defining how the DNA rearrangements contribute to genetic disease becomes more challenging. Often, the pathogenic consequence of these mutations involves disruption of regulatory genomic landscapes that, in turn, alters spatiotemporal gene expression (Spielmann et al., 2018; Sánchez-Gaya et al., 2020).

### Cis-Regulatory Elements: Dictating When, Where and How Much a Gene Is Expressed

Cis-regulatory elements (CREs) are sequences of non-coding DNA controlling the expression of nearby genes (Li et al., 2015). Enhancers are the most abundant (Shen et al., 2012) and most well-studied of the distal CREs (Ray-Jones and Spivakov, 2021). Enhancers increase the expression of their target gene(s), often by recruiting transcriptional machinery and making physical contact with the target promoter (Gasperini et al., 2020; Panigrahi and O’Malley, 2021). Enhancer-promoter (EP) interactions can show marked spatiotemporal specificity and may span large genomic distances, with reports of enhancers located up to 1.45 Mb away from their target gene (Long et al., 2020). This remarkable specificity was elegantly demonstrated in a series of experiments investigating the regulation of the developmental gene *SOX9*, which identified two novel enhancer clusters regulating *SOX9* expression within a narrow developmental window (Long et al., 2020). Strong chromatin contacts between the novel enhancers and the *SOX9* promoter were observed in cranial neural crest cells, but not in their embryonic stem cell precursors (Long et al., 2020). Furthermore, these enhancers were inactive in cranial chondrocytes which are directly derived from cranial neural crest cells, and interestingly, also display robust *SOX9* expression (Long et al., 2020). Accordingly, genes with tightly controlled expression profiles often utilize multiple enhancers that can act in a cooperative or even redundant manner (Spitz, 2016; Moorthy et al., 2017). Therefore, whilst we are now able to predict the genomic location of putative enhancers by the presence of distinct epigenetic signatures (Bulger and Groudine, 2011), inferring their target gene(s) and their spatiotemporal activity profile presents an enduring challenge (Pennacchio et al., 2013).

In contrast, silencers are regions of DNA that repress the expression of their target gene(s) (Maston et al., 2006) and represent an understudied and poorly understood class of CREs (Gisselbrecht et al., 2020; Pang and Snyder, 2020). However, by systematically screening genomic fragments for repressive activity, over 5,000 candidate human silencers have been recently discovered and were found to share many properties of enhancers such as tissue specificity and long-range communication (Pang and Snyder, 2020). Intriguingly, the use of a *Drosophila* model recently identified a surprisingly large number of ‘bifunctional elements’, i.e., regulatory sequences that can function as *either* enhancers or silencers, depending upon the spatiotemporal context, further attesting to the complexity of gene regulation (Gisselbrecht et al., 2020).

Another important class of distal CREs are insulator elements. These are often binding sites for ‘insulator proteins’ such as CCCTC-binding factor (CTCF) (Bell et al., 1999). Insulators have a key role in segmenting the genome into discrete structural units which largely define the genomic region over which regulatory elements can act (Bushey et al., 2008). This both facilitates the formation of appropriate long-range regulatory interactions, whilst also preventing aberrant communication between CREs and non-target genes (Geyer and Clark, 2002; Kuhn and Geyer, 2003). Insulators can also function as barriers that prevent the spread of heterochromatin into adjacent euchromatic regions of DNA (Gaszner and Felsenfeld, 2006). Thus, insulators have a clear impact on the nuclear organization of chromatin (Phillips-Cremins and Corces, 2013).

### The Role of the 3D Genome in Transcriptional Regulation

Transcriptional regulation heavily relies on the spatial organization of chromatin within the three-dimensional nucleus. Addressing the many complexities of 3D chromatin architecture is beyond the scope of this review, however, excellent articles provide in-depth discussion of this topic (Bonev and Cavalli, 2016; Stadhouders et al., 2019).

**FIGURE 3:**
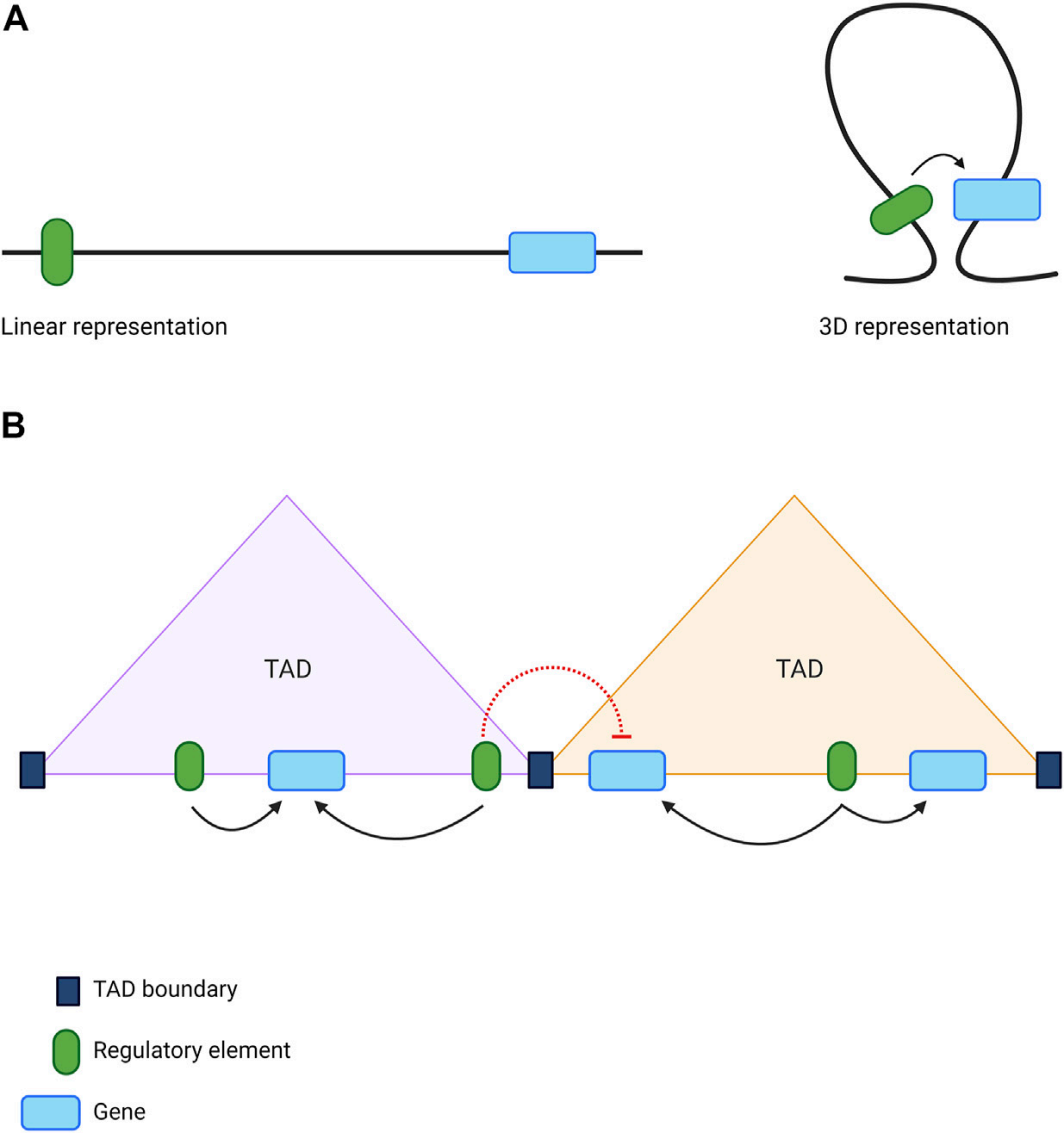
The role of 3D genome organisation in long-range gene regulation **(A)** The folding of chromatin brings distant sequences into close proximity enabling the formation of long-range interactions between genes and distal regulatory elements **(B)** The genome is organised into topologically associating domains (TADs), which act as local regulatory hubs. Sequences of DNA located within the same TAD can interact frequently with each other (black arrows), while chromatin interactions spanning TAD boundaries are much less common (red dotted lines). This restricts the activity of regulatory elements to the appropriate genes. Image created with BioRender.com.

The functionality of CREs is intimately dependent upon chromatin structure (Kleinjan and van Heyningen, 2005). For example, enhancers located within inaccessible, nucleosome-dense regions of chromatin are deemed silent (Bozek and Gompel, 2020), while histone modifications can be used to infer whether an enhancer is in an active or poised state (Creyghton et al., 2010; Rada-Iglesias et al., 2011; Zentner et al., 2011). Furthermore, interactions between distal CREs and target genes can span regions of linear DNA that are very distant from one another (Lettice et al., 2003). These long-range interactions, essential for gene regulation, are largely mediated by the folding of chromatin into hierarchical 3D structures known as ‘topologically associating domains’ (TADs) (Figure 3A) (Dixon et al., 2012; Nora et al., 2012; Rao et al., 2014). Regions of DNA within the same TAD interact frequently with each other, whilst inter-TAD interactions are much less commonly observed (Dixon et al., 2012). TADs are largely conserved between cell types and across species (Dixon et al., 2012), although tissue specificity is observed at the level of sub-TADs; smaller and more dynamic domains visible at the sub-megabase scale (Phillips-Cremins et al., 2013). These structural domains represent local regulatory landscapes, with insulator elements often demarcating the boundary that separates two adjacent TADs (Figure 3B) (Dixon et al., 2012; Rao et al., 2014; Schoenfelder and Fraser, 2019).

Here, we have provided a simplified overview of long-range gene regulation, which is a highly complex, multi-faceted process and remains an active area of research. Despite significant progress in recent years, our current understanding of long-range gene regulation remains incomplete (Schoenfelder and Fraser, 2019). In some cases, for example, experimentally depleting TAD boundaries produces a surprisingly minimal impact on gene expression (Nora et al., 2017; Rao et al., 2017), indicating that additional mechanisms are at play. However, it is clear that SV are capable of altering local regulatory domains and inducing pathogenic changes in transcriptional regulation (Kleinjan and Coutinho, 2009; Lupiáñez et al., 2016; Spielmann et al., 2018).

## The Disease-Associated Insertions at Xq27.1 can Disrupt Long-Range Gene Regulation

The genomic landscape of the Xq27.1 locus has shown the disease-associated insertions occur within a gene desert. Therefore, the pathomechanism of these complex mutations does not involve disrupting the sequence of known coding genes located within this genomic region. However, the insertions introduced at Xq27.1 often contain regions of DNA that encode either partial (Bowl et al., 2005; Zhu et al., 2011; Chou, 2012; DeStefano et al., 2013; Bunyan et al., 2014; Brewer et al., 2016; Middelkamp et al., 2019) and/or full length (Zhu et al., 2011; Haines et al., 2015) genes, although three of the insertions consist entirely of non-coding sequence (Taylor et al., 2015; Si et al., 2019; Boschann et al., 2022). As the reported insertions are unbalanced, the over-expression of the duplicated genes (either full length or partial transcripts) may be a potential pathomechanism (Bowl et al., 2005; Bunyan et al., 2014; Brewer et al., 2016). This possibility has often been deemed unlikely for multiple reasons including 1) the insertion lacks an open reading frame (Bowl et al., 2005), 2) the duplicated genes have not been associated with the relevant disease phenotype (Haines et al., 2015), or 3) over-expression of the inserted gene(s) was not observed in quantitative gene expression experiments (Brewer et al., 2016; Gaynor et al., 2020).

Another possible pathomechanism may involve aberrant splicing between the inserted sequence and an adjacent gene, resulting in the formation of a fusion transcript (Bunyan et al., 2014; Brewer et al., 2016). In the case of CMTX3, this is unlikely as the inserted sequence and the nearest downstream gene are transcribed from opposite strands (Brewer et al., 2016). This mechanism has not been directly tested for the other insertions at Xq27.1, although *in silico* splice site prediction for the rearrangement associated with congenital ptosis suggests that it cannot be excluded as a possibility (Bunyan et al., 2014). Mechanisms involving aberrant splicing are more likely for those insertions containing truncated transcripts with an intact transcription start site but lacking transcription termination signals. In a similar manner, the formation of aberrant read-through transcripts has been observed in other diseases where deletions remove the 3’ portion of a gene, which results in transcriptional read-through into the neighbouring gene and epigenetic promoter silencing (Tufarelli et al., 2003; Ligtenberg et al., 2009).

Among researchers reporting the insertions at Xq27.1, the most common hypothesis proposed to explain the pathogenicity involves transcriptional dysregulation of nearby genes (Bowl et al., 2005; Zhu et al., 2011; Chou, 2012; DeStefano et al., 2013; Haines et al., 2015; Taylor et al., 2015; Brewer et al., 2016; Middelkamp et al., 2019; Si et al., 2019; Boschann et al., 2022). SV can pathologically alter regulatory landscapes via multiple mechanisms, with recent reviews available for a comprehensive commentary (Kleinjan and Coutinho, 2009; Spielmann and Mundlos, 2013; Krijger and De Laat, 2016; Lupiáñez et al., 2016; Cutrupi et al., 2018; Spielmann et al., 2018; D’haene and Vergult, 2020; Sánchez-Gaya et al., 2020).

One possibility is that the large insertions result in a position effect on nearby gene(s), altering spatiotemporal regulation by physically distancing the gene from its typical CREs (DeStefano et al., 2013). Initially, this was postulated as a potential pathomechanism for XL-CGH, with reports of three unique interchromosomal insertions ranging from 126—389 kb causing a common clinical phenotype of excessive hair growth (DeStefano et al., 2013). However, this view has repeatedly been challenged as additional pathogenic insertions were identified at the same genetic locus giving rise to very different clinical phenotypes. Some of the additional insertions reported were significantly larger and would therefore be expected to produce a similar position effect (Haines et al., 2015). Whilst the disconnection of genes and regulatory elements is a known pathomechanism of SV (Spielmann et al., 2018), the current evidence suggests this is not a common mechanism driving the various diseases associated with insertions into the Xq27.1 locus (Si et al., 2019).

Transcriptional dysregulation can also arise from mutations that disrupt TAD boundaries, given that long-range regulatory interactions typically occur within the constraints of these defined genomic domains (Lupiáñez et al., 2015). According to publicly available Hi-C data, the pathogenic insertions at Xq27.1 occur within a TAD and are therefore unlikely to disrupt boundary sites (Brewer et al., 2016; Gaynor et al., 2020). However, the disruption of sub-TAD boundaries may be a possibility and could contribute to the tissue-specificity of the disease phenotypes. Rather than disturbing pre-existing boundaries, Gaynor and others (2020) suggested that the insertions could instead introduce ectopic boundary elements into Xq27.1 which may alter local regulatory interactions. Such a disease mechanism is relatively understudied, however a recent investigation by Willemin and others (2021) has provided valuable insights. After generating a transgenic mouse line whereby a TAD boundary element usually located on chromosome two was inserted into a large TAD on chromosome 10, the authors observed splitting of the host TAD into two smaller regulatory domains which was associated with tissue specific changes in local gene expression (Willemin et al., 2021).

Enhancer (or silencer) deletions should also be considered as potential pathomechanisms for the diseases caused by complex genomic rearrangements, such as those observed in XL-HPT, where the insertions occur in combination with deletions at Xq27.1. Interchromosomal insertions from 2p25.3 accompanied by deletions at Xq27.1 were reported in two unrelated XL-HPT kindreds (Bowl et al., 2005; Taylor et al., 2015). The inserted fragments do not share common sequence despite originating from the same chromosome 2p25.3 sub-band, however the deletions overlap by ∼ 1.5 kb (Gaynor et al., 2020). As the deleted sequence did not contain ultraconserved elements, which often act as tissue-specific enhancers (Pennacchio et al., 2006), CRE deletion was considered an unlikely disease mechanism (Gaynor et al., 2020). Ultraconserved elements, however, account for only a subset of CREs (Visel et al., 2008) with many enhancers displaying species specificity (Bulger and Groudine, 2011), and therefore this hypothesis should not be discounted.

One of the more prevalent hypotheses postulates that the insertions may introduce ectopic CREs into Xq27.1 which can interact with nearby gene(s) and alter spatiotemporal expression profiles (Zhu et al., 2011; Haines et al., 2015; Middelkamp et al., 2019; Si et al., 2019). Multiple lines of evidence lend support to this hypothesis. Firstly, the nature of the inserted sequence seems important in dictating the phenotype, since the various insertion breakpoints occur within the same palindromic sequence (Si et al., 2019), and insertions of comparable size are associated with different clinical phenotypes. For example, a ∼126 kb insertion causes hypertrichosis (Zhu et al., 2011) while a ∼120 kb insertion is associated with bilateral ptosis (Bunyan et al., 2014). Furthermore, by consulting publicly available annotations of the non-coding genome, multiple researchers have identified putative regulatory elements residing within the inserted fragments (Middelkamp et al., 2019; Si et al., 2019). Interestingly, a super-enhancer implicated in craniofacial development is contained within an insertion at Xq27.1 that has been classified as a VUS in a patient with a complex congenital phenotype which includes craniofacial anomalies (Middelkamp et al., 2019). Ectopic enhancer adoption is a disease mechanism that has been observed in similar genomic rearrangements. A recent study using the Hammer toe mouse model, which displays a syndactyly phenotype, identified a large interchromosomal insertion occurring upstream of the sonic hedgehog (*Shh*) gene which contains enhancer elements that drive ectopic *Shh* expression during limb development (Mouri et al., 2018).

Irrespective of the underlying molecular mechanism, transcriptional dysregulation of nearby genes has been observed in studies investigating three of the disease-associated insertions at Xq27.1 (DeStefano et al., 2013; Haines et al., 2015; Brewer et al., 2016). Lymphoblasts isolated from XX male sex reversal patients displayed ectopic expression of *SOX3* (Haines et al., 2015), whilst XL-CGH patient keratinocytes showed aberrant regulation of *FGF13* (DeStefano et al., 2013). Dysregulation of *FGF13* was also observed in lymphoblasts derived from CMTX3 patients (Brewer et al., 2016). Clearly, the pathogenic rearrangements are capable of disrupting local regulatory landscapes and altering the expression profiles of nearby genes. It is interesting to note that both *SOX3* and *FGF13* are developmental regulator genes (Si et al., 2019) with important roles in mammalian embryogenesis (Rizzoti et al., 2004; Puranam et al., 2015). In general, developmental regulator genes are disproportionately affected in ‘cis-ruption diseases’, i.e., genetic conditions caused by altered cis-regulation (Kleinjan and Coutinho, 2009). Developmental genes typically carry out multiple functions within a variety of cell types at specified time points, and as such, their expression is usually controlled by the action of numerous spatiotemporally specific CREs (Kleinjan and Coutinho, 2009; Spitz and Furlong, 2012; Spielmann et al., 2018). The marked complexity of their regulatory landscapes, combined with the dosage sensitivity of these developmental genes, may render them less tolerant to disruptions in the local regulatory region (Kleinjan and Coutinho, 2009).

It is clear that SV can act via multiple, intriguing pathomechanisms, including some that are not discussed here (e.g. disturbing non-coding RNA genes, reviewed in D’haene and Vergult, (2020)). Whilst we cannot assume that all of the insertions are acting in a similar manner, there is sufficient evidence to suggest that local gene dysregulation may be a recurrent mechanism amongst this group of diseases.

## Difficulties in Identifying the Candidate gene(s) Being Dysregulated by the Disease-Associated Insertions

Identifying candidate genes being dysregulated by the disease-associated insertions at Xq27.1 has proven a challenging task for many of the phenotypes studied. This is not an uncommon problem as predicting the consequences of complex SV is notoriously difficult and remains an active area of research. This is partly because large SV can simultaneously affect multiple genes and CREs. In some instances, the phenotype can be explained by the activity of a single dysregulated gene, however other phenotypes may arise from the combined effects of the mutation on several candidates (Weischenfeldt et al., 2013; Middelkamp et al., 2019). Long-range gene regulation can span large genomic distances and therefore, many genes could be considered as potential candidates. For example, when investigating gene dysregulation for XL-CGH, the expression level of all genes located within 3 Mb either side of the causative SV were analyzed (DeStefano et al., 2013). Predicting the effect of complex SV is made more challenging by the incomplete annotation of the non-coding genome. Despite the concerted effort and progress in recent years, obtaining a comprehensive, genome-wide catalogue of CREs remains a challenging task (Pennacchio et al., 2013). Although bioinformatic tools are useful in predicting the presence of putative CREs, the data cannot adequately inform on important aspects of functionality, including 1) specific target gene(s) directly regulated by the CRE, and 2) the spatiotemporal context in which the CRE is active/inactive (Pennacchio et al., 2013).

In an attempt to overcome many of these challenges, Middelkamp and others (2019) proposed a systematic pipeline for solving SV pathomechanisms that combines bioinformatics with experimental methods, whilst also considering the patient’s unique phenotype. This approach was used to investigate a range of complex SV found in patients with genetically unsolved (neuro)developmental disorders. Briefly, potential candidate genes (within or 2 Mb adjacent to the SV), were ranked based on phenomatch scores calculated by comparing the patient’s phenotype to known phenotypes already associated with each gene (Middelkamp et al., 2019). Transcriptome analyses were then performed using peripheral blood cells to assess candidate gene expression (Middelkamp et al., 2019). To account for the shortcomings of using blood cells to investigate gene dysregulation driving (neuro)developmental disorders, the authors utilized numerous computational tools to further assess candidate genes, including data from multiple cell types at every stage of analysis. Using this strategy, Hi-C datasets predicted genes most likely to be impacted by the mutation (Middelkamp et al., 2019). Epigenomic datasets identified nearby enhancers and predicted how many were being displaced by the SV for each candidate gene (Middelkamp et al., 2019). Promoter capture Hi-C (PCHi-C) data was then queried to detect known long-range interactions being disrupted by the mutation (Middelkamp et al., 2019). This comprehensive pipeline provides an innovative approach to solving this complex problem and is particularly well-suited to identifying pathomechanisms that involve disruptions to known chromatin interactions (Middelkamp et al., 2019). However, it has been hypothesized that the disease-associated insertions at Xq27.1 may instead cause disease through introducing novel CREs into the local genomic region, resulting in aberrant expression of nearby gene(s) (Zhu et al., 2011; Haines et al., 2015; Si et al., 2019). If so, predicting which gene(s) form ectopic interactions with the novel CREs will be important in understanding the disease mechanism, yet is a daunting task given our limited understanding of long-range gene regulation. This pipeline makes great use of publicly available datasets to investigate local regulatory landscapes. However, some of the relevant technologies, such as PCHi-C, were only recently developed, with data available from a limited number of cell types. Although PCHiC data from 22 cell types was analyzed in this pipeline, 18 of these originate from a hematopoietic lineage. Since regulatory interactions can show marked cell-type specificity, the relevance of publicly available datasets to the diseases associated with insertions at Xq27.1 is questionable, and this data may need to be experimentally obtained.

We also encourage readers to consider that the phenotypic consequences of non-coding mutations can be distinct from coding mutations affecting the same gene (Kleinjan and van Heyningen, 2005). This is clearly observed for the developmental gene, *SHH*. Coding mutations in *SHH* cause holoprosencephaly (Roessler et al., 1996), whilst mutations disrupting an *SHH* enhancer cause the limb-specific phenotype of preaxial polydactyly (Lettice et al., 2003). Phenomatching is useful for prioritizing potential candidate genes, although using it as a filtering strategy may result in important candidates being overlooked.

Due to the multiple disease mechanisms that may underly SV, combined with the incomplete understanding of gene regulation, functional assays are still needed to validate the predictions that arise from these pipelines. Although not highly feasible for large patient cohorts, functional validation will require personalized, rather than high-throughput approaches to account for the patient genetic backgrounds and the specific cell-types impacted by each disease phenotype.

## Importance of Using Disease-Relevant Tissue to Investigate the Disease-Associated Insertions at Xq27.1

The diseases caused by insertions at Xq27.1 show marked tissue-specificity. Patients with CMTX3, for example, display an isolated peripheral neuropathy phenotype (Huttner et al., 2006). Therefore, to properly address the prevailing hypothesis that gene dysregulation underlies these complex phenotypes, it is essential that functional studies are performed in the affected tissue type relevant to each disease.

This was clearly demonstrated by previous investigations into the ∼389 kb insertion that causes XL-CGH (DeStefano et al., 2013). Dysregulated expression of *SOX3,* the closest gene to the pathogenic insertions, was initially considered the most likely mechanism underlying this disease (Zhu et al., 2011). However, transcriptomic analysis of patient skin biopsies identified the dysregulation of another nearby gene, *FGF13*, in XL-CGH patients relative to controls (DeStefano et al., 2013). Further analysis revealed that patient keratinocytes displayed a 6.7-fold reduction in *FGF13* mRNA, whereas expression in fibroblasts was comparable to controls (DeStefano et al., 2013). These findings from different tissues harboring the ∼389 kb insertion clearly demonstrate that the structural variants at Xq27.1 can produce cell-type specific dysregulation. Whilst XL-CGH affects cell types that are easily obtainable from patients (skin cells), this is not true for many other diseases associated with insertions at Xq27.1. Without studying disease-relevant tissue, determining the effect of these mutations on the local gene regulation remains an enduring challenge.

When describing the respective insertions at Xq27.1, the *SOX3* gene is often proposed as the most likely candidate given its proximity to the pathogenic insertions (∼82 kb) and its important role in developmental regulation (Bowl et al., 2005; Zhu et al., 2011; Taylor et al., 2015; Middelkamp et al., 2019; Boschann et al., 2022). However, experimentally investigating this hypothesis has proven very challenging. In many cases, researchers have been unable to detect *SOX3* expression when using readily available tissue types, such as peripheral blood samples (Si et al., 2019), skin biopsies (DeStefano et al., 2013), and lymphoblasts (Brewer et al., 2016). This is perhaps unsurprising as this gene belongs to the SOXB1 family of transcription factors that are widely expressed in the early vertebrate embryo and developing nervous system (Uchikawa et al., 1999; Wood and Episkopou, 1999; Lefebvre et al., 2007). Access to disease-relevant tissue has not been possible for some of the phenotypes described, including XL-HPT, which affects embryonic parathyroid gland development (Gaynor et al., 2020). Furthermore, modelling these large and complex chromosomal rearrangements using available cloning technologies is currently not feasible. Thus, to investigate the role of *SOX3* in XL-HPT, Gaynor and others (2020) generated knockout animal models with targeted deletions of either *SOX3* or a nearby ultraconserved element (uc482) presumed to regulate *SOX3* expression. For both transgenic lines, null hemizygotes displayed normal parathyroid gland development and function, suggesting that XL-HPT involves spatiotemporal disturbances in *SOX3* expression that cannot be recapitulated by a knockout model (Gaynor et al., 2020).

Overall, research into the disease-associated insertions at Xq27.1 has provided some valuable lessons for investigating the consequences of intergenic SV including: 1) it is important, although often difficult, to perform studies in the appropriate cellular context, which can depend on both cell-type and developmental stage, and 2) it is critical to utilize the patient’s genetic background to retain the pathogenic rearrangement, as generic animal knockout models are not reflecting the regulatory disruptions causing these specific diseases. Fortunately, recent developments in cellular reprogramming technologies can now provide access to patient-derived disease-relevant tissue, thereby circumventing the major challenges that have previously led to a research impasse for these disorders.

## Cellular Reprogramming Technology Provides Access to Disease-Relevant Tissue

Induced pluripotent stem cells (iPSC) theoretically have the capacity to differentiate into any somatic cell population and can be generated from readily accessible sources such as fibroblasts (Takahashi et al., 2007), peripheral blood (Loh et al., 2009), and urine samples (Zhou et al., 2012). Robust protocols are available that utilize iPSC to generate cell types implicated in the pathogenesis of multiple diseases associated with insertions at Xq27.1. CMTX3, for example, can now be studied by differentiating patient-derived iPSC into the major cell types of the peripheral nervous system, including motor neurons (Du et al., 2015), sensory neurons (Saito-Diaz et al., 2021), and Schwann cells (Kim et al., 2017). Alternative reprogramming technologies may also be used to obtain neural tissue, such as ‘direct lineage conversion’ or ‘direct reprogramming’ approaches (e.g., Liu et al., 2016; Kim et al., 2020). In both approaches, the desired cell type is generated from mature somatic cells using a protocol that bypasses a pluripotent intermediate population (Prasad et al., 2016). Direct reprogramming produces multipotent progenitors, whilst direct lineage conversion does not (Prasad et al., 2016). Whilst these technologies appear an attractive and time-efficient option, there are advantages of using iPSC-based approaches for studying the diseases associated with insertions at Xq27.1.

iPSC reprogramming reverts mature cells back to a pluripotent embryonic state (Lapasset et al., 2011), and subsequent differentiation protocols attempt to mirror *in vivo* developmental processes to produce the desired cell type (Zhu and Huangfu, 2013). For example, motor neuron differentiation mimics signaling pathways that occur in the developing spinal cord, guiding iPSC through multiple, increasingly specialized precursor populations including caudal neuroepithelial cells and motor neuron progenitors (Du et al., 2015). Access to embryonic cell types is invaluable considering that many of the diseases associated with insertions at Xq27.1 display a clear developmental etiology. This includes, but is not limited to, XX male sex reversal, characterized by abnormal gonad development (Haines et al., 2015), and XL-HPT, involving agenesis of the parathyroid glands (Bowl et al., 2005) (see Table 1 for additional developmental phenotypes). Fortunately, it is now possible to generate bipotential gonad/testis-like cells (Knarston et al., 2020) and parathyroid-like cells (Lawton et al., 2020) from human iPSC, providing unprecedented access to tissue types that are relevant for studying these complex developmental disorders. Cells can be analyzed at multiple timepoints throughout differentiation, enabling researchers to investigate the dynamic regulatory processes governing tissue development, and the impact that intergenic SV may have on this defined process. As previously discussed, *SOX3* is a high-priority candidate gene likely to be impacted by the insertions and has already been implicated in the pathogenesis of XX male sex reversal (Haines et al., 2015). Given that *SOX3* encodes a transcription factor involved in embryonic development, investigating developmental cell types will provide a valuable insight into how the disease-associated insertions may alter the spatiotemporal regulation of this interesting candidate gene.

Despite their utility, there are some inherent limitations to iPSC models that should be considered. Experimental variability can arise from multiple sources including genetic background of the donor (Rouhani et al., 2014), donor cell-type (Kim et al., 2011) and laboratory-specific practices (Volpato et al., 2018). iPSC lines can vary in their propensity to differentiate into certain lineages (Bock et al., 2011; Boulting et al., 2011). This must be considered when using iPSC models to investigate the disease-associated insertions at Xq27.1. Developmental anomalies are observed in several of these diseases, and the high-priority candidate gene, *SOX3,* is a transcription factor that has been shown to regulate differentiation in studies from numerous animal models (Bylund et al., 2003; Dee et al., 2008; Bergsland et al., 2011; McAninch et al., 2020). Aberrant differentiation may therefore be a cellular phenotype in patient-derived iPSC, and if so, it will be important to disentangle disease-related differences in differentiation from background line-line variability. Donor genetic background accounts for most of the variability observed between iPSC lines (Kajiwara et al., 2012; Rouhani et al., 2014; Kyttälä et al., 2016), and can be avoided through the use of isogenic controls. Unfortunately, the large, complex nature of the pathogenic insertions at Xq27.1 limits the capacity to generate isogenic control lines, in which case, parental controls may be considered instead (Sánchez-Gaya et al., 2020). Several publications propose additional strategies for minimizing the effects of variability, including the use of relevant statistical approaches and a careful experimental design (Germain and Testa, 2017; Volpato and Webber, 2020). Although iPSC offer a unique opportunity to investigate cellular development *in vitro*, they cannot precisely mirror the complexity of embryogenesis (Sánchez-Gaya et al., 2020). However, co-cultures and 3D organoid systems are becoming increasingly useful for establishing cellular interactions and creating 3D microenvironments that more closely resemble *in vivo* development (Shi et al., 2017; Lee et al., 2020). Despite these limitations, iPSC technology is increasingly being recognized as a valuable tool for studying the pathogenic consequences of complex SV (Drakulic et al., 2020; Sánchez-Gaya et al., 2020). Recently, a patient-derived iPSC model was used to identify the specific regulatory disturbance causing branchio-oculo-facial disorder in a patient harboring a large chromosomal inversion (Laugsch et al., 2019). To date, this approach has not been applied to investigate any of the disease-associated insertions at Xq27.1, yet will likely be an important tool for uncovering the mechanistic complexities of these intriguing phenotypes.

## Experimental Approaches for Solving the Pathomechanism of CMTX3

**FIGURE 4:**
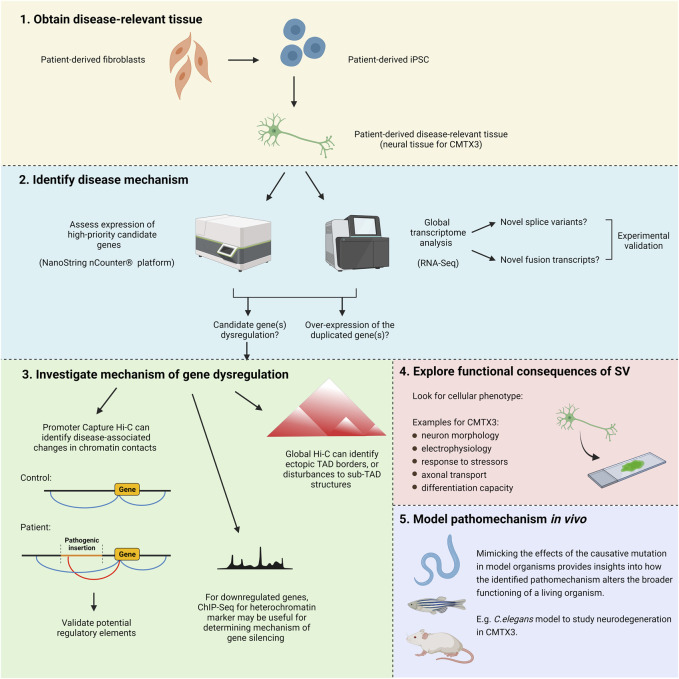
Experimental approach for uncovering the pathogenic mechanism of CMTX3 and the other diseases associated with insertions(+/-deletions) at Xq27.1. (1) Induced pluripotent stem cells retaining the patient genetic background can be used to generate patient-derived disease-relevant tissue for downstream analysis. (2) The expression level of high-priority candidate genes can be specifically assessed through NanoString nCounter^®^ analysis. Global transcriptomic analysis can be carried out using RNA-Seq. In addition to the predominant hypothesis of gene dysregulation, this approach directly addresses alternative pathomechanisms previously hypothesised to underly these diseases, such as abnormal splice variants, aberrant fusion transcripts, and overexpression of the duplicated genes. (3) If gene dysregulation is observed, experiments can help to uncover the underlying mechanism. Promoter Capture Hi-C can identify alterations in long-range regulatory interactions. Global Hi-C can identify ectopic TAD boundaries that might be introduced by the insertions, as well as changes to tissue-specific sub-TAD organisation. If the disease mechanism involves gene repression, ChIP-Seq for a heterochromatin marker (H3K9me3) can identify whether this arises from mutation-induced alterations to the local chromatin environment rather than altered regulatory interactions (Laugsch et al., 2019). (4) A range of functional tests can be performed to look for a cellular phenotype in patient samples. (5) Potential pathomechanisms can be studied *in vivo* using model organisms. Image created with BioRender.com.

After obtaining disease-relevant tissue and performing *in silico* predictions, experimental tools can be used to functionally explore the mechanisms underlying complex SV. Recent publications have clearly demonstrated the utility of iPSC-based approaches for revealing the pathomechanism of these types of mutations (Laugsch et al., 2019; Sánchez-Gaya et al., 2020). In this review, we will expand upon and modify some of these methodologies to create a pipeline suitable for investigating CMTX3 and the other disease-associated insertions at Xq27.1 (Figure 4). However, this approach could also be useful in guiding research into similar rearrangements located elsewhere in the genome.

Local gene dysregulation is a potential pathomechanism driving CMTX3. Earlier research demonstrated that the causative insertion alters the regulation of *FGF13* in patient lymphoblasts (Brewer et al., 2016). However, since this was not assessed in neuronal tissue, it remains unclear whether aberrant *FGF13* regulation is directly implicated in the pathogenesis of CMTX3 or merely a bystander effect of this complex SV within lymphoblasts (Brewer et al., 2016). Therefore, the first step of our experimental pipeline will address gene expression analysis within patient-derived neuronal tissue.

The expression level of positional candidate genes (i.e., those contained within, or 3 Mb adjacent to, the pathogenic insertion) can be specifically assessed through targeted transcriptome profiling. By utilizing tools such as the NanoString Technologies nCounter^®^ platform, custom panels can be designed to accurately quantify expression of high priority candidates at the mRNA and/or protein level. Importantly, by screening samples from progressive time-points throughout differentiation, it is possible to identify gene dysregulation that may be temporally restricted.

Global transcriptome profiling using RNA-seq should also be performed on CMTX3 neuronal tissue. This will provide an additional dataset to validate dysregulation of candidate genes, and through gene ontology analysis, can identify specific biological pathways impacted by the SV. Importantly, RNA-seq analysis will also detect fusion transcripts and/or novel transcript variants arising from the inserted fragment, which are alternative pathomechanisms that may cause CMTX3.

Once a strong candidate gene (or genes) has been identified, experimental tools can help uncover the molecular mechanisms driving the dysregulation. Using the candidate gene(s) promoter as an anchor, Capture Hi-C (Mifsud et al., 2015; Schoenfelder et al., 2015), can identify the specific regions of chromatin that interact with the target gene(s). Comparing the interaction profiles of patient and control samples will facilitate the identification of any disease-associated changes in the 3D chromatin contacts (i.e., gains or losses of DNA interactions). The genomic coordinates participating in the differential interactions should then be queried in genome browsers to gain an insight into potential disease mechanisms. For example, epigenomic datasets (e.g. Ensembl Regulatory Build; Zerbino et al., 2015) can be used to predict whether the region encodes putative CREs, which would suggest that altered regulatory interactions may underly the disease. Ideally, data from disease-relevant tissue should be utilized, although this may not be possible if the candidate gene dysregulation is spatially and temporally restricted. If the relevant tissue is not available, epigenomic data can instead be experimentally obtained. By using ChIP-Seq to profile various histone modifications, as well as ATAC-Seq to identify regions of open chromatin, novel enhancers regulating *SOX9* expression within iPSC-derived cranial neural crest cells were recently predicted (Long et al., 2020). Any potential CREs identified using these techniques should be functionally validated, which can be achieved using a variety of *in vitro* and/or *in vivo* reporter assays (Laugsch et al., 2019; Long et al., 2020). A global Hi-C approach may also be useful, particularly for determining whether ectopic TAD boundaries are being introduced by the pathogenic insertion, and for further characterizing the local regulatory landscape within the disease-relevant tissue. Micro-C, a modern variant of Hi-C technology, utilizes micrococcal nuclease (MNase) rather than restriction enzymes to fragment the genome (Hsieh et al., 2015), allowing for a much higher resolution view of chromatin organization. With the capacity to detect ∼three to five fold more chromatin loops than conventional Hi-C, Micro-C improves the identification of individual regulatory interactions whilst providing a deeper insight into global chromatin architecture (Krietenstein et al., 2020).

Gene dysregulation may also arise through position effect variegation, whereby a gene can be silenced if it is placed in the vicinity of heterochromatin (Kleinjan and Van Heyningen, 1998). If the pathomechanism appears to involve candidate gene repression, ChIP-Seq analysis for the heterochromatin marker, H3K9me3, can be useful for determining whether the downregulation is arising from inappropriate heterochromatinization of the local region rather than altered regulatory interactions (Laugsch et al., 2019).

Morphological and functional characterization of patient-derived cells will provide deeper insight into the biological processes causing the disease. Having an understanding of the underlying pathomechanism will be useful for prioritizing the types of phenotypic tests to perform. Furthermore, assessing gene dysregulation in the context of global transcriptomic analysis can assist in identifying cellular pathogenic readouts. As an example, RNA-Seq analysis identified dysregulation of genes involved in cellular migration in samples obtained from a patient harboring a complex SV causing branchio-oculo-facial syndrome (Laugsch et al., 2019). Subsequently, *in vitro* migration assays were performed, revealing an impaired migratory capacity in patient-derived cranial neural crest cells (Laugsch et al., 2019). After identifying likely pathomechanisms, model organisms can be used to assess how these diseases more broadly affect animal function and behavior *in vivo*. The nematode *Caenorhabditis elegans* (*C. elegans*) is particularly useful for modelling neuronal diseases, including CMT, due to their simple and well-characterized nervous system. Recently, another form of X-linked CMT, CMTX6, was modelled using this approach, which was able to recapitulate the locomotion deficits and neurodegeneration presenting in patients (Narayanan et al., 2021). Whilst CMTX6 is caused by a point mutation (Kennerson et al., 2013), *C. elegans* is also suitable for modelling pathomechanisms associated with complex SV, such as gene dysregulation. Gene upregulation can be studied with an overexpression model, whereby tissue-specific promoters or inducible expression systems can confer spatiotemporal specificity. RNA interference of endogenous gene orthologues can model candidate gene downregulation, which may also be restricted to desired cell-types (Qadota et al., 2007) or developmental stages (Sin et al., 2014). Furthermore, alternative pathomechanisms such as novel splice variants or fusion transcripts can be studied via the delivery of transgenic constructs.

## Conclusion

We have proposed a strategy for investigating the pathomechanism underlying CMTX3 and the other diseases associated with similar genomic rearrangements occurring at chromosome Xq27.1. We have incorporated the use of cutting-edge technologies that have a demonstrated capacity to revolutionize our understanding of complex SV, but have not yet been applied to study the disease-associated insertions (+/- deletions) at Xq27.1. This intriguing group of diseases may act as a unique paradigm for providing valuable insight into the pathogenic mechanisms underlying complex intergenic SV and more broadly, enhance our understanding of long-range gene regulation. Overall, SV involving the non-coding genome represents an understudied class of mutation contributing to unsolved genetic diseases that have been excluded for gene coding mutations. Understanding the molecular mechanisms will be essential for improving genetic diagnosis and guiding the development of future therapies for patients suffering from these debilitating conditions.
